# Outcomes of first-line treatment and their association with pretreatment neutrophil-to-lymphocyte ratio in patients with advanced renal cell carcinoma: Insights from a tertiary care institute in Pakistan

**DOI:** 10.3332/ecancer.2024.1753

**Published:** 2024-09-03

**Authors:** Mirza Rameez Samar, Maha Javaid, Nida e Zehra, Nawazish Zehra, Muhammad Arif Hameed, Misbah Younus Soomro, Insia Ali, Yasmin Abdul Rashid

**Affiliations:** 1 Medical Oncology, Department of Oncology, The Aga Khan University, Karachi 74600, Pakistan; 2 Department of Oncology, The Aga Khan University, Karachi 74600, Pakistan

**Keywords:** renal cell carcinoma, progression-free survival, first-line treatment, metastatic, NLR

## Abstract

**Background:**

Renal cell carcinomas (RCCs) are renal parenchymal neoplasms that contribute to <5% of cancer cases worldwide. Within the diverse group of renal tumours, clear cell carcinoma is the most common subtype. The recommended first-line treatment for metastatic disease is a tyrosine kinase inhibitor given either as monotherapy or in combination with an immune checkpoint inhibitor, based on improved survival outcomes. These endpoints are not only influenced by the initial risk stratification but also by certain variables such as the neutrophil-to-lymphocyte (NLR) ratio.

**Methods:**

A retrospective review was conducted to evaluate the progression-free survival (PFS) with first-line treatment in patients with metastatic RCC treated at our institute from the year 2017–2021. We also investigated the association of PFS with both Memorial Sloan Kettering Cancer Center risk groups and the pretreatment NLR ratio.

**Results:**

Overall, 35 patients were enrolled after fulfilling the eligibility criteria. Of these, 25 patients received Pazopanib, 5 patients were treated with Sunitinib and the remaining patients were administered Pembrolizumab with Axitinib. Two-thirds of the study population belonged to the intermediate-risk group. The median PFS for all participants was 16 months. Among the overall population, patients in the favourable-risk group demonstrated superior PFS. Patients with elevated pretreatment NLR experienced shorter PFS compared to the patients with low to normal NLR.

**Conclusion:**

This review highlights the prognostic significance of initial risk stratification and pretreatment NLR in predicting the response to first-line treatment in metastatic RCC patients. As this is a comprehensive study emphasizing the outcomes of metastatic RCC in Pakistan, it fills a void in the literature by providing invaluable perspectives on the real-world outcomes of patients. This not only enhances our understanding of disease management in this region but also lays the foundation for future investigations.

## Background

Kidney cancer refers to the neoplasm originating from the renal parenchyma. It is included among the ten most common cancers diagnosed in men and women combined, comprising 4.1% of all newly diagnosed cancer cases [1]. In the United States, approximately 76,000 new cases are diagnosed annually and almost 14,000 deaths occur from kidney cancer each year. According to the American Cancer Society, the global incidence has risen by 2% per year over the past two decades. Despite this rise, the 5-year survival rate has decreased by 1% per year and is currently 75% for all stages combined [2]. This decline can be mainly attributed to the increased use of imaging modalities, which has led to the early detection of incidental renal tumours. According to GLOBOCAN 2020, renal cell carcinoma (RCC) constitutes up to 1.5% of all cancers diagnosed in Pakistan [3]. In a 5-year observational study, more than 4,000 patients were diagnosed with kidney cancer in Pakistan [4].

Clear cell carcinoma (ccRCC) is the major histological subtype, accounting for 70–80% of RCCs [5]. In a Pakistani study conducted by Latif *et al* [6], ccRCC contributed to 73.1% of kidney cancers. Other subtypes include papillary carcinoma, chromophobe tumours, urothelial carcinoma of the renal pelvis, oncocytoma, collecting duct tumours and renal sarcomas. RCC is more frequent in men than in women with a ratio of 1.7:1. Most people diagnosed are older, with an average age of 64 years. Approximately one-quarter of such patients are found to have de novo metastatic disease, whereas around 20%–40% of those with localised disease are prone to develop metastasis later in their lifetime [7]; the most common sites being lung, lymph nodes, bone and liver [8]. In a review published by Mohsin *et al* [9] 63% of the patients in Pakistan presented with localised renal tumours confined to the kidneys.

Since the introduction of Memorial Sloan Kettering Cancer Center (MSKCC) scoring system, patients with metastatic RCC have been classified into three risk groups: favourable, intermediate and poor. Over the last decade, medical treatment has transitioned from an era entailing the use of interferons, interleukins and targeted therapies against the vascular endothelial growth factor (VEGF) pathway to the age of immune checkpoint inhibitors (ICIs) against program cell death-1 or its ligands. This is largely due to the limited efficacy of both traditional chemotherapy and radiotherapy in RCC [10]. Even with these advancements, disparities in the cost of these therapies play a significant role in deciding the first-line treatment in Pakistan. While several studies have focused on the management of RCCs from a surgical standpoint, there remains a clear gap in data from Pakistan regarding the available systemic therapies for metastatic RCC and their outcomes.

In recent times, neutrophil-to-lymphocyte ratio (NLR), a biological marker, has emerged with potential prognostic implications in various malignancies including kidney cancer. Although its role has been primarily studied in localised RCCs to predict recurrences after nephrectomy [11–15], fewer studies have explored NLR as a means to predict survival in metastatic disease [16, 17].

In this study, we aim to determine the progression-free survival (PFS) to first-line treatment in patients with metastatic RCC presenting to a tertiary care institute in Pakistan. Furthermore, we also aim to differentiate these patients by their PFS, based on MSKCC and pretreatment NLR.

## Materials and methods

We conducted a retrospective review involving patients diagnosed with metastatic RCC between 2017 and 2021, at The Department of Oncology, The Aga Khan University Hospital, Karachi. The tumour was classified as per the TNM staging system (8th edition 2017). All patients received treatment at The Aga Khan University Hospital. Patients with either localised disease or sarcomatoid histology were excluded. Confidentiality of the patients was maintained by assigning predefined serial numbers instead of using medical record numbers. Patients were then stratified into risk groups as per the MSKCC score.

The response was documented according to the Response Evaluation Criteria in Solid Tumours by contrast-enhanced computed tomography of the chest, abdomen and pelvis done every 12 weeks of treatment. Disease response was defined as an interval reduction in either the size of the primary lesion and/or the size and number of metastatic deposits. Progressive disease was documented by the interval development of new lesions or an increase in the size of previous metastatic deposits. The NLR was calculated from the patient’s full blood count by dividing the absolute neutrophil count by the absolute lymphocyte count. The number 3 was taken as the cutoff value, with values greater than 3 considered high, whereas values up to 3 considered low. Clinical data was obtained from electronic or paper records. The study was conducted following approval from the institutional review board of The Aga Khan University Hospital.

### Statistical analysis

The data were entered and analysed using the Statistical Package for Social Science version 20 (Chicago, Illinois). Categorical variables were analysed for frequency, percentage and graphical representations. For continuous data, we used an independent sample *T* test. Categorical data were analysed using Fisher’s exact test. Kaplan–Meier survival curves were generated to present patients’ PFS, and the log-rank test was utilised to compare median PFS times.

## Results

### Baseline demographics and clinical characteristics

A total of 35 patients fulfilled the eligibility criteria of the study. The average age of the patients was 55 years. Of the study participants, 4 (11%) were female and 31 (88%) were male. More than 60% of patients had ECOG status of 1 whereas only 2 patients (5.7%) had ECOG status of 3. Comorbidities were present in all the patients. The most prevalent comorbid conditions were hypertension (42%), diabetes (37%) and ischemic heart disease (17%). Up to one-third of the study participants had ccRCC whereas histopathology in one-fifth of the participants did not reveal any variant. The mean hemoglobin level, as reported by the laboratory parameters of all the patients, was 11.8 gm/dL, with a standard deviation (SD) of +1.83. The mean lymphocyte count and mean neutrophil count were 1.67 and 4.23, respectively. The mean platelet count was 303 × 10^3^. The mean lactate dehydrogenase and calcium levels were 271 and 8.5, respectively. Most of the participants belonged to the intermediate risk group as per the MKSCC grouping system (Table 1).

### Treatment details

The majority of patients (85%) received tyrosine kinase inhibitor (TKI) monotherapy as first-line treatment. Twenty-five (71.4%) participants received Pazopanib as first-line treatment. Of these, more than one-third received Pazopanib at a dose of 400 mg once daily. Among five patients who received Sunitinib, more than half of the patients tolerated a dose of 37.5 mg every 4 of 6 weeks. Among 35 patients, dose adjustment was needed in 23 (65.7%) participants. The most common reason for dose adjustment was asthenia, found in 12 patients (34.3%) (Table 2).

### Progression-free survival

**Figure 1.**
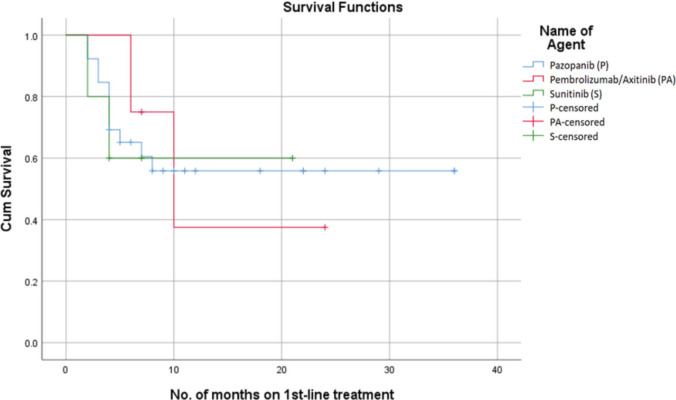
PFS on first-line treatment.

Among the three treatment groups, Pazopanib had the most notable impact on PFS, with a probability of survival exceeding 55% at 22 months. However, Sunitinib showed a 60% probability of PFS at 22 months, while the combined use of Pembrolizumab with Axitinib had a 38% probability of PFS at 22 months (Figure 1). The median PFS for the overall population was 16 months (95% confidence interval: 4.01–20.98). The mean PFS in patients who received Pazopanib was 22 + 4.5 months. In comparison, patients treated with Sunitinib had a mean PFS of 13.8 + 3.7 months, while those who received Pembrolizumab with Axitinib had a mean PFS of 14.2 + 4.2 months.

### Correlation analysis of PFS with NLR

**Figure 2.**
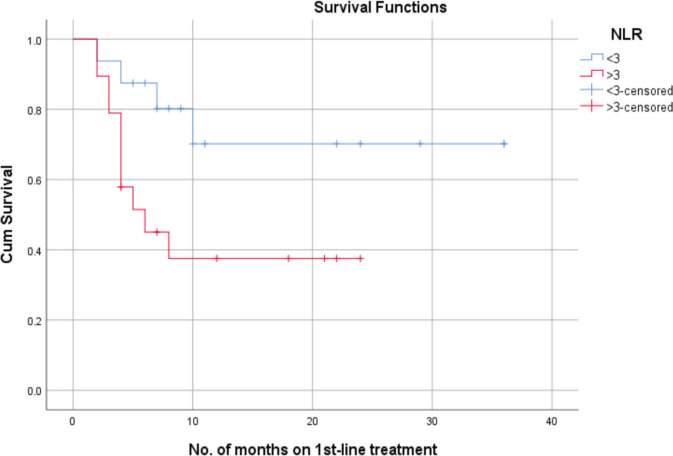
PFS as per NLR.

Patients with an NLR of less than 3 exhibited a mean PFS of 27 + 5.1 months. In contrast, those with an NLR of 3 or higher had a mean PFS of 11.6 + 2.3 months. In correlation analysis, those with an NLR under 3 had a 70% probability of PFS at 24 months, compared to less than 40% for those with an NLR over 3 (Figure 2).

### Correlation of PFS with MSKCC subgroups

**Figure 3.**
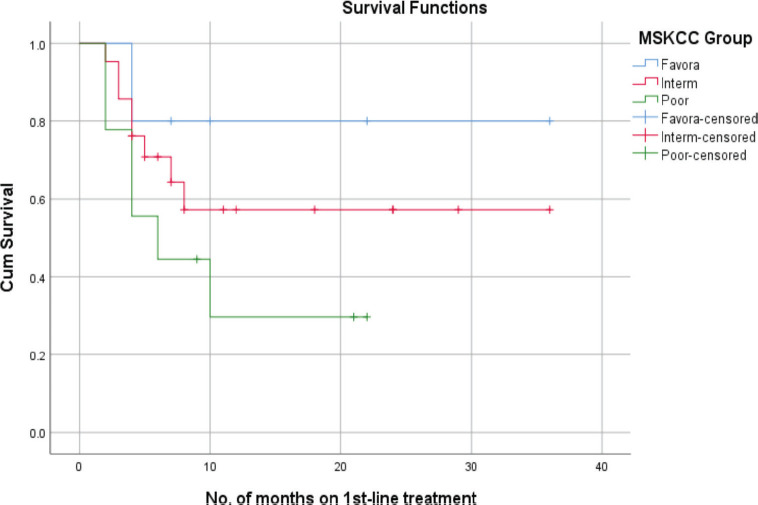
PFS of RCC patients with MSKCC.

According to the MSKCC subgroups, the mean PFS for the favourable-risk group was 29 + 5.3 months, whereas the mean PFS for the intermediate-risk and poor-risk groups were 22 + 4.6 and 10 + 3.1 months, respectively. In a correlation analysis, the favourable-risk group showed an 80% probability of survival at 22 months, while the probability of survival for the intermediate-risk and poor-risk groups, were 58% and 30% at 22 months, respectively (Figure 3).

## Discussion

Kidney cancer originating from the renal parenchyma can involve one of two major structures, outer cortex or inner medulla. Approximately a quarter of such patients are found to have locally advanced or metastatic disease upon diagnosis [7]. To date, various agents have been approved in the first-line setting for metastatic RCC. With ICI now incorporated into first-line treatment regimens, there has been a notable increase in median PFS rates – from 5.6 months in the era of targeted therapy to 23.9 months when combined with ICI [18–22]. Nevertheless, the 5-year survival rate remains as low as 13% [1].

In this review, we aimed to determine the PFS of first-line treatment and its association with MSKCC risk criteria as well as pretreatment NLR in patients with metastatic RCC treated at a tertiary care hospital in Karachi, Pakistan. In our study, most of the patients (77.1%) were diagnosed with de novo metastatic disease, with the lungs as the most frequently involved organ, followed by lymph nodes, bones and liver. The order of metastatic spread observed in our review aligns with the findings from a prospective study by Erman *et al* [23] which demonstrated a similar sequence of metastatic involvement. In a study evaluating the effectiveness of first-line TKIs in 40 Indian patients with metastatic RCC, the median PFS was observed to be 10.8 months across the entire cohort [24]. In this analysis, most of the participants were male, aged over 50 years, with the majority having an ECOG score of 1 and classified as intermediate-risk according to MSKCC criteria. Similarly, the participants in our study exhibited these same characteristics.

Our study reported a median PFS of 16 months with first-line treatment. This is in line with the median PFS found in the study conducted by Rini *et al* [25] which investigated the combination of ICI and TKI in the first-line setting. Likewise, in a phase 3 trial investigating first-line Nivolumab and

Cabozantinib in metastatic RCC, the median PFS was found to be 16.6 months, which closely matches the PFS reported in our review [26]. Given the economic constraints and limited access to newer therapies, the majority of our patients were treated with a single-agent TKI, reflecting the standard practice in our region at that time. While several studies on first-line VEGF-targeted therapy have reported median PFS ranging from 7.09 to 11.5 months [23, 24, 27–32], a prospective study found a median PFS of 10 months with first-line Pazopanib [23]. Among the participants, 19% were long-term responders, with their responses extending up to 18 months. The slightly improved PFS observed in our review is likely due to the predominant use of Pazopanib among our patients, along with the small number of patients receiving combined ICI and TKI therapy, both of which may have contributed to the better PFS.

Numerous prognostic models have been developed to correlate the survival of patients with metastatic RCC. Among these, the MSKCC risk classification is the most widely used criterion, first introduced in 1999 by Motzer *et al* [33] which involved stratifying patients into three risk groups. These risk groups not only differ from each other with respect to the presence or absence of adverse factors but also in terms of survival outcomes. In a retrospective analysis of 2,390 patients, the median PFS of the favourable risk group was found to be superior to that of the intermediate and poor-risk groups [34]. In a comparable study by Heng *et al* [35] the 2-year overall survival rates were reported as 75% for the favourable risk group, 53% for the intermediate risk group and 7% for the poor risk group. A similar trend was also depicted in our study, where the favourable risk group outperformed with the highest PFS rate of 80% at 22 months, followed by the intermediate risk group with a PFS rate of 58% during the same timeframe, and then the poor-risk group, which showed decreasing rates thereafter. This sequence of differences in PFS is consistent with that observed in previous studies [36, 37].

Additionally, the prognosis of metastatic RCC is also affected by other factors such as the NLR, which is a biomarker of systemic inflammation that has demonstrated prognostic correlation in different malignancies including RCC [38–43]. The data are diverse and there is a lack of consensus on a specific value for classifying patients as having high or low NLR, with most studies using 3 as the cut-off value [16, 44, 45]. In a study conducted by Hu *et al* [46] patients with a pretreatment NLR value of >3 were found to have a shorter PFS compared to those with an NLR of <3. Likewise, we observed a negative correlation between elevated pretreatment NLR levels above 3 and PFS, corroborating the results from various meta-analyses [47, 48]. Furthermore, this effect was observed regardless of the first-line therapy used, as highlighted in a systematic review by Shao *et al* [49].

Our study had few limitations. First, this was a retrospective analysis. Second, it was a study confined to a single center. Therefore, these results cannot be generalised to the population of this region. Third, the small sample size may have affected the primary outcome, especially considering the limited number of patients in the favourable-risk and poor-risk groups, which may not accurately reflect the actual influence of these risk groups on PFS. Fourth, this study was conducted during a phase, when the use of ICI was getting started in our region. Furthermore, given that our country is recognised as a ‘lower-middle income country’ by the World Bank, the majority of our patients were unable to pursue ICI due to financial constraints, leaving TKI as the primary first-line treatment option for our patients.

## Conclusion

This study not only sheds light on the significance of initial risk stratification and pretreatment NLR but also provides essential insights into the prognosis and treatment of metastatic RCC in Pakistan. As this is the first study from this region highlighting the systemic treatment of metastatic RCC in the current era of ICIs, it lays the groundwork for future research in this field from this region. With the evolving landscape of systemic therapies, there is an urgent need for prospective multicenter studies to validate these findings and identify additional variables that may influence treatment outcomes in this patient population.

## Conflicts of interest

The authors declare that they have no competing interests.

## Funding

No specific funding has been used for manuscript writing or reporting.

## Consent for publication

Not applicable.

## Ethics approval and consent to participate

This study was approved by the Institutional Ethics Review Committee (ERC number: 2021-6684-19963).

## Availability of data and materials

The data that support the findings of this study are available from the corresponding author upon reasonable request.

## Author contributions

MRS, MJ and YAR were instrumental in shaping the initial manuscript. Notably, MRS took a central role in conceptualising, refining the format and enhancing the final rendition. MAH and MS earnestly participated in reviewing and providing crucial input. YAR and IA meticulously examined the concluding draft. All authors unanimously endorsed the final version for publication. NEZ and NZ adeptly conducted data analysis, contributing their statistical expertise. MRS played a key role in the final manuscript submission process.

## Figures and Tables

**Table 1.** Demographic table.

Variables	*N* = 35 (100%)
**Age** **Mean (SD)**	55 (+12.2)
**Gender** **Male** **Femal**e	31 (88.6%) 4 (11.4%)
**ECOG** **1** **2** **3**	22 (62%) 11 (31%) 2 (5.7%)
**Comorbid illness** **Hypertension** **Diabetes** **Ischemic** h**eart** d**isease** **Osteoporosis** **Chronic** k**idney** d**isease** **Chronic** l**iver** d**isease** **Chronic** o**bstructive** p**ulmonary** d**isease** **Hypothyroidism** **Benign** p**rostatic** h**yperplasia**	15 (42%) 13 (37.1%) 06 (17%) 1 (0.02%) 1 (0.02%) 1 (0.02%) 1 (0.02%) 1 (0.02%) 1 (0.02%)
**Presenting** c**omplaint** **Shortness of breath** **Flank pain** **Painless hematuria** **Abdominal pain** **Lower limb pain** **Incidental finding** **Backache**	10 (28.6%) 07 (20%) 07 (20%) 05 (14.3%) 03 (8.6%) 02 (5.7%) 02 (2.6%)
**Metastatic** d**isease** **De novo** **Recurrence**	27 (77.1%) 08 (22.9%)
**Site of** m**etastasis** **Lung** **Lymph nodes** **Bone** **Liver** **Brain**	30 (85.6%) 14 (40%) 08 (22.8%) 07 (20%) 02 (5.7%)
**Presence of thrombosis** **Yes** **No**	5 (14.3%) 30 (85.7%)
**Nephrectomy performed** **Yes** **No**	13 (37.1%) 22 (62.9%)
**Histologic subtype￼** **Clear cell** **Papillary** **Unclassified**	24 (69%) 04 (11%) 07 (20%)
MKSCC subgroup Favourable Intermediate Poor	05 (14%) 21 (60%) 09 (26%)
**Mean** h**ematological** p**arameters (SD)** **Hemoglobin** **Platelets** **Neutrophil** c**ount** **Lymphocyte** c**ount** **NLR** **Lactate dehydrogenase** **Calcium**	11.8 (+1.83) 303 (+111.2) 4.23 (+2.0) 1.67 (+0.75) 0.55 (+0.50) 271 (+164.6) 8.5 (+2.35)

**Table 2.** Treatment details table.

Variables	N = 35 (100%)
**1st-line treatment options** **ICI + TKI** TKI	5 (14.3%) 30 (85.7%)
**1st-line agents** **Pazopanib** **Pembrolizumab + Axitinib** **Sunitinib**	25 (71.4%) 5 (14.3%) 5 (14.3%)
**Dose of Pazopanib** **400 mg once daily** **600 mg once daily** **800 mg once daily**	12 (34.3%) 5 (14.3%) 8 (22.9%)
**Dose of Sunitinib** **37.5 mg once daily every 4/6 weekly** **50 mg once daily every 4/6 weekly**	3 (8.6%) 2 (5.7%)
**Dose adjustment needed** **No** **Yes**	12 (34.3%) 23 (65.7%)
**Reason for dose adjustment** **Asthenia** **Diarrhea** **Diarrhea Hand-foot syndrome** **Deranged liver functions** **Thrombocytopenia** **Anemia**	12 (52.2%) 4 (17.4%) 3 (13.1%) 2 (8.7%) 1 (4.3%) 1 (4.3%)
